# Dietary Deoxynivalenol (DON) May Impair the Epithelial Barrier and Modulate the Cytokine Signaling in the Intestine of Atlantic Salmon (*Salmo salar*)

**DOI:** 10.3390/toxins10090376

**Published:** 2018-09-14

**Authors:** Torfinn Moldal, Aksel Bernhoft, Grethe Rosenlund, Magne Kaldhusdal, Erling Olaf Koppang

**Affiliations:** 1 Norwegian Veterinary Institute, Post box 750 Sentrum, 0106 Oslo, Norway; torfinn.moldal@vetinst.no (T.M.); aksel.bernhoft@vetinst.no (A.B.); magne.kaldhusdal@vetinst.no (M.K.); 2 Skretting ARC, Post box 48, 4001 Stavanger, Norway; grethe.rosenlund@skretting.com; 3 Department of Basic Sciences and Aquatic Medicine, Norwegian University of Life Sciences, Post box 369 Sentrum, 0102 Oslo, Norway

**Keywords:** atlantic salmon, deoxynivalenol, feed, intestine, PCR, proliferating cell nuclear antigen, suppressor of cytokine signaling, tight junctions

## Abstract

Impaired growth, immunity, and intestinal barrier in mammals, poultry, and carp have been attributed to the mycotoxin deoxynivalenol (DON). The increased use of plant ingredients in aquaculture feed implies a risk for contamination with mycotoxins. The effects of dietary DON were explored in 12-month-old Atlantic salmon (*Salmo salar*) (start weight of 58 g) that were offered a standard feed with non-detectable levels of mycotoxins (control group) or 5.5 mg DON/kg feed (DON group). Each group comprised two tanks with 25 fish per tank. Five fish from each tank were sampled eight weeks after the start of the feeding trial, when mean weights for the control and DON groups were 123.2 g and 80.2 g, respectively. The relative expression of markers for three tight junction proteins (claudin 25b, occludin, and tricellulin) were lower, whereas the relative expression of a marker for proliferating cell nuclear antigen was higher in both the mid-intestine and the distal intestine in fish fed DON compared with fish from the control group. The relative expression of markers for two suppressors of cytokine signaling (SOCS1 and SOCS2) were higher in the distal intestine in fish fed DON. There was no indication of inflammation attributed to the feed in any intestinal segments. Our findings suggest that dietary DON impaired the intestinal integrity, while an inflammatory response appeared to be mitigated by suppressors of cytokine signaling. A dysfunctional intestinal barrier may have contributed to the impaired production performance observed in the DON group.

## 1. Introduction

Atlantic salmon (*Salmo salar*) is one of the most important species in aquaculture worldwide. Salmonids are carnivores by nature, but raw materials of vegetable origin are increasingly used in feed for farmed salmonids due to a limited supply of fish meal and fish oil [1,2,3]. The use of cereals in feeds for farmed fish implies a risk for contamination with mycotoxins that are metabolites of mold capable of having acute toxic, carcinogenic, mutagenic, teratogenic, immunotoxic, or hormonal effects in mammals [4]. Deoxynivalenol (DON) is one of the major mycotoxins produced by *Fusarium* spp. [5]. The current European Union (EU) legislation establishes 8000 μg/kg as a maximum guidance value for DON in cereals and cereal products (with an exception for maize by-products) intended for animal feed and 5000 μg/mg as a maximum guidance value for complementary and complete feeding stuffs [6,7]. The surveillance of feeds for salmonids in Norwegian aquaculture shows levels of mycotoxins far below the current guidance levels [8], but accidental intermixture may occur.

Mycotoxins do not appear to pose a hazard to the consumer, because carry-over is considered negligible in fillets from gilthead seabream (*Sparus aurata*) and Atlantic salmon experimentally exposed to dietary DON at levels up to 5.5 mg/kg feed [9,10]. However, adverse effects in fish ingesting feed contaminated by mycotoxins have been observed. In Atlantic salmon and rainbow trout (*Oncorhynchus mykiss*), there appears to be a linearity between increasing dietary levels of DON up to 5.5 mg/kg feed and 2.6 mg/kg feed, respectively, and decreasing weight gain and growth rate [11,12]. Furthermore, dose-related alterations in some blood parameters were shown in Atlantic salmon and rainbow trout [11,13], and pathological changes in the liver characterized by subcapsular hemorrhage and edema were observed in a number of DON-exposed rainbow trout [12]. In carp (*Cyprinus carpio*), the relative expression of markers for both pro- and anti-inflammatory cytokines as well as enzymes in different organs including the intestine were activated by feed-borne DON at a concentration of 953 μg/kg feed with an adaption over time [14].

The intestine is an important barrier for protection against pathogens in addition to its main task in digestion. Exposure of the intestine to mycotoxins, either via feed or gavage, may impair the intestinal integrity and consequently alter the absorption of nutrients as well as facilitate the invasion of microbes. In pigs exposed to dietary DON at levels from 0.9 to 3.5 mg/kg feed, the relative expression of markers for several tight junction proteins (TJs) and inflammatory markers in intestines were affected [15,16,17]. The relative expression of markers for certain cytokines was down-regulated in the intestines of broiler chickens on a diet contaminated with 10 mg DON/kg feed [18]. Furthermore, DON predisposes to the development of necrotic enteritis in broiler chickens at a contamination level of 3 to 4 mg/kg feed [19].

Dose-dependent up-regulation of pro-inflammatory cytokines, followed by up-regulation of suppressors of cytokine signaling and subsequent basal expression of the cytokines, has been demonstrated in several organs in mice orally exposed to DON over a dose range of 0.1–12.5 mg/kg body weight in short-time experiments [20]. Altered transcript levels as well as protein expression for TJs, decreased transepithelial electrical resistance, and increased permeability as a result of exposure to DON have been observed in several in vitro and in vivo models indicating functional effects [21].

So far, little is known about the impact of dietary mycotoxins on intestinal health of fish in general and of Atlantic salmon in particular. The aim of this study was to investigate in Atlantic salmon the long-term impact of dietary DON at a level of 5.5 mg/kg feed on parameters related to the epithelial barrier of the intestine (relative expression of markers for tight junction proteins and proliferating cell nuclear antigen as well as goblet cell density), cytokine-mediated inflammation (relative expression of a marker for interleukin 1β), and suppression of cytokine signaling (relative expression of markers for SOCS1 and SOCS2), as well as morphologic responses to intestinal insults. The chosen DON level was slightly higher than the maximum recommended level (5 mg DON/kg feed) established in the European Union’s current legislation on animal feed.

## 2. Results

### 2.1. Gene Expression Analysis by Real-Time PCR

**Figure 1:**
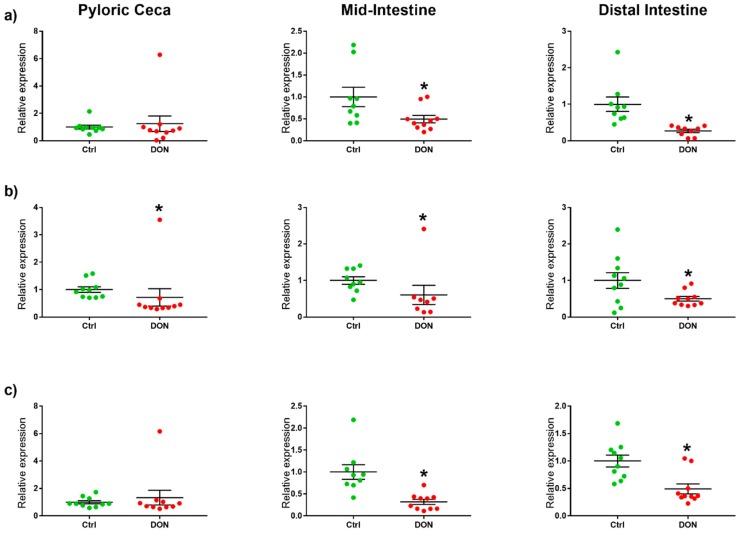
Relative expression of markers for tight junction proteins in the intestine of salmon fed deoxynivalenol (DON) (5.5 mg/kg feed) or no DON (controls) for eight weeks. * Significant differences (unpaired *t*-test or Mann–Whitney U test, *p* < 0.05) between the experimental groups in the same intestinal segment. The relative expression of markers for the tight junction proteins (**a**) claudin 25b, (**b**) occludin, and (**c**) tricellulin were significantly lower in both the mid-intestine and the distal intestine from fish fed DON compared with the controls. The relative expression of the marker for occludin was also significantly lower in the pyloric ceca from fish fed DON compared with the controls.

The relative expression of markers for the tight junction proteins claudin 25b, occludin, and tricellulin was significantly lower in both the mid-intestine and the distal intestine from fish fed DON compared with fish from the control group (see Table 1 and Figure 1). The relative expression of the marker for occludin was also lower in the pyloric ceca from fish fed DON compared with the controls.

**Figure 2:**
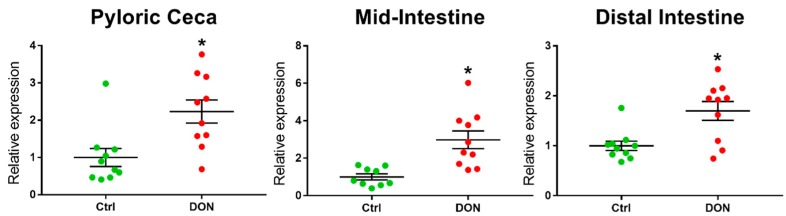
Relative expression of a marker for proliferating cell nuclear antigen (PCNA) in the intestine of salmon fed DON (5.5 mg/kg feed) or no DON (controls) for eight weeks. * Significant differences (unpaired *t*-test or Mann–Whitney U test, *p* < 0.05) between the experimental groups in the same intestinal segment. The relative expression of a marker for PCNA was significantly higher in all intestinal segments from fish fed DON compared with the controls.

On the other hand, the relative expression of a marker for proliferating cell nuclear antigen (PCNA) was significantly higher in all intestinal segments in fish fed DON compared with the controls (see Table 1 and Figure 2).

**Figure 3:**
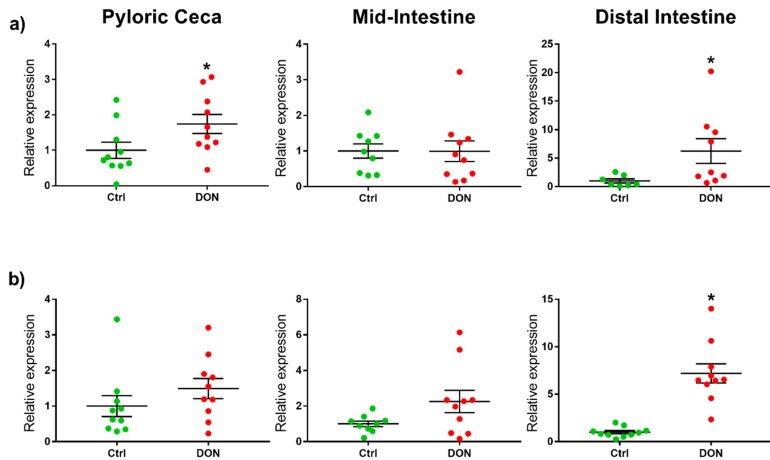
Relative expression of suppressors of cytokine signaling (SOCS) in the intestine of salmon fed DON (5.5 mg/kg feed) or no DON (controls) for eight weeks. * Significant differences (unpaired *t*-test or Mann–Whitney U test, *p* < 0.05) between the experimental groups in the same intestinal segment. The relative expression of markers for the suppressors of cytokine signaling (**a**) SOCS1 and (**b**) SOCS2 were significantly higher in the distal intestine from fish fed DON compared with the controls. The relative expression of the marker for SOCS1 was also significantly higher in the pyloric ceca from fish fed DON compared with the controls.

The relative expression of markers for the suppressors of cytokine 1 and 2 (SOCS1 and SOCS2) was significantly higher in the distal intestine in fish fed DON compared with the controls. Further, the relative expression of the marker for SOCS1 was also significantly higher in the pyloric ceca in fish fed DON compared with the controls (see Table 1 and Figure 3).

There was no significant difference between the dietary groups regarding the relative expression of a marker for the cytokine interleukin 1β in any of the intestinal segments (see Table 1).

### 2.2. Histology and Immunohistochemistry

**Figure 4:**
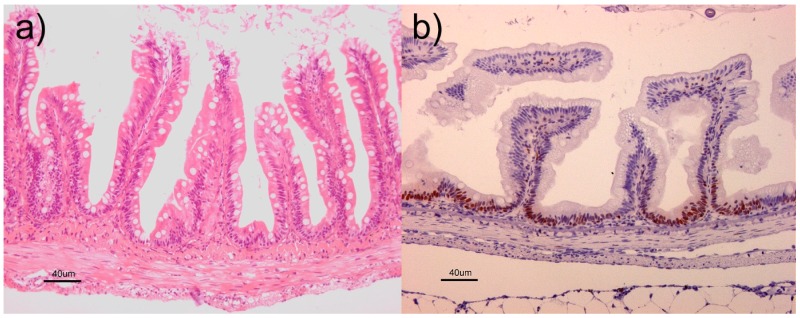
Micrographs of intestinal tissue. Following a histological evaluation, (**a**) pathological changes were not revealed in the intestines of any fish and (**b**) proliferating cells were detected mainly at the base of the folds with an equal pattern in the controls and the DON group.

Histological examination did not reveal pathological changes in the intestines of any fish (see Figure 4a). The polarization of the enterocytes seemed non-affected, the density of goblet cells appeared similar in both dietary groups, while proliferating cells were mainly detected at the base of the intestinal folds with an equal pattern for both groups (see Figure 4b).

## 3. Discussion

We found that 5.5 mg DON/kg feed was associated with reduced intestinal expression of markers for three tight junction proteins (claudin 25b, occludin, and tricellulin), suggesting insufficient expression of these proteins, which could lead to an epithelial barrier impairment with sequelae affecting both structure and function of the intestine. The effect was most prominent in the distal intestine. An assumed functional impairment was confirmed by the fact that feed intake and feed efficiency were significantly reduced by DON exposure [11], and the body weight gain of the DON group was only one third of the weight gain of the fish from the control group. It was therefore surprising that we did not detect indications of intestinal inflammation (expression of a marker for IL-1β and histological examination) or proliferation of goblet cells (tissue sections stained with Alcian Blue (AB) and Periodic acid–Schiff (PAS) in combination) and absorptive epithelial cells (tissue sections stained with an antibody toward proliferating cell nuclear antigen (PCNA)). However, other findings suggested a subtler mechanism behind the adverse impact of DON on production performance. We detected that the relative expression of markers for two suppressors of cytokine signaling proteins (SOCS1 and SOCS2) was increased. These increased levels may have contributed to the suppression of inflammatory cytokines and morphologic inflammatory responses. We also found that the intestinal level of an mRNA marker for PCNA was increased by DON, suggesting an attempt at increased proliferation of epithelial cells. However, for some unknown reason this gene transcription did not appear to translate into the appropriate protein.

To the best of our knowledge, this is the first study addressing the possible influence of any mycotoxin on certain parameters of the intestine of Atlantic salmon (*Salmo salar*). The relative expression of markers for three tight junction proteins (TJs) was significantly lower in both the mid-intestine and the distal intestine in fish experimentally exposed to dietary DON for eight weeks compared with fish fed a standard diet with non-detectable levels of mycotoxins. These findings suggest that the intestinal permeability was increased leading to a leakage of fluids and a suboptimal control of paracellular influx of macromolecules as undigested food particles, pathogens, and toxins. Possible consequences include reduced growth as observed in this trial [10,11] and a number of infections both locally and systemically. The relatively high expression of the marker for PCNA in all intestinal segments in fish exposed to DON compared with the controls may be interpreted as a local response aimed at regenerating intestinal integrity. In pigs experimentally exposed to DON in the feed, both the up- and down-regulation of markers for TJs have been reported [15,16]. This apparent response discrepancy might be attributed to different dosages and study periods.

In carp (*Cyprinus carpio*), the relative expression of markers for several cytokines was 2–3-fold higher in the intestine in fish on an experimental diet with DON at a level of 953 μg/kg feed compared with the controls at day 14 of the experiment, but the relative expression returned to basal levels at day 26 and day 56 [14]. In mice exposed to DON by oral gavage, both the relative expression of markers for tumor necrosis factor-α and interleukin 6 as well as the protein expression were rapidly induced in several organs and plasma, respectively, with a peak of 2 h after exposure and a subsequent decrease [20]. However, the relative expression of several suppressors of cytokine signaling (SOCS), a family of intracellular proteins that play critical roles in the regulation of innate and adaptive immune responses as well as growth and development through negative feedback on cytokine signaling, remained high for several hours with different kinetics for each SOCS and organ [22]. Functional conservation between teleosts and mammals for SOCS has been demonstrated [23], and the relatively high expression of markers for SOCS1 and SOCS2 in the distal intestine in Atlantic salmon exposed to dietary DON in our study, in combination with the absence of inflammation, suggest a successful and long-lasting response to DON in orchestrating the complex network of cytokines. Specifically, SOCS1 is central to the regulation of a number of cytokines including interferons (IFNs) and interleukins in mammals [22], and a strong and negative regulatory effect of salmon SOCS1 on type I and type II IFN-signaling has been demonstrated in cells originating from Atlantic salmon head kidney [23]. Salmon SOCS2 was only moderately affected by IFN responses in that study; however, given the analogy with SOCS2 in mammals [22], it can be assumed that in our study, SOCS2 has influenced growth hormone activity and thus contributed to the reduced growth in fish exposed to DON compared with the controls [10,11]. These findings underline the importance of taking into consideration the dynamics and complexity of cytokine signaling when designing studies and interpreting results.

The level of DON in the feed for the DON group was slightly higher than the maximum recommended level (5 mg DON/kg feed) established in the European Union’s current legislation on animal feed. The surveillance of feeds for salmonids in Norwegian aquaculture shows in general low levels of mycotoxins [8], but a negative impact on certain biochemical parameters have already been observed in Atlantic salmon at 2 mg DON/kg feed [11].

Previous studies have produced findings suggesting that the distal intestine (also designated as the second segment of the mid-intestine and posterior intestine) is more vulnerable to disease and dysfunction and particularly important for the immunological defense of Atlantic salmon [24,25,26]. These works showed that the uptake of gold-labelled bovine serum albumin was restricted to the distal intestine and that the transcript levels of selected immune parameters in general were higher in this intestinal segment, where an inflammation associated with soy-bean meal also occurs in this species. Our results agree with the results from these studies as DON appeared to impair the epithelial barrier and modulate the cytokine signaling in the distal intestine to a larger degree than the other intestinal segments examined.

Studies applying real-time PCR have demonstrated that markers for several tight junction proteins, among these claudin 25b, occludin, and tricellulin, are expressed in the intestine of Atlantic salmon [27,28], and that the expression levels are elevated when juvenile Atlantic salmon are transferred to seawater, suggesting that they are involved in the reorganization of intestinal epithelium and have possibly changed the paracellular permeability. Studies on the protein expression of junctional proteins to reveal whether there is an association between gene expression and protein expression would be of great interest. Unfortunately, research in Atlantic salmon is hampered by the lack of relevant antibodies for performing immunohistochemistry or western blot. However, our findings demonstrate that DON-containing feed may influence certain parameters in the intestine in several ways that separately and in combination may have an adverse impact on fish growth and health. Because the increased use of raw materials of plant origin in aquaculture feed implies a risk for contamination of mycotoxins, the continued surveillance of feeds for the presence of mycotoxins as well as more research exploring the effects of mycotoxins on health and productivity are needed.

## 4. Materials and Methods

### 4.1. Animal Ethics and Rearing

The feed trial took place at the Skretting ARC Lerang Research Station that is approved by the Norwegian Animal Research Authority. The trial was approved by the responsible person for animal ethics at the facility on 12 April 2011 and carried out in accordance with the recommendations in the current animal welfare regulations in Norway (FOR-1996-01-15-23).

The trial is described in detail elsewhere [10,11]. Briefly, juvenile post-smolt Atlantic salmon (*Salmo salar*) (SalmoBreed, Bergen, Norway, 12 months old, both genders) with an average weight of approximately 58 g were randomly allocated into tanks supplied by flow-through seawater. All the fish were vaccinated intraperitonally against *Aeromonas salmonicidae* three weeks after the start of the experiment.

### 4.2. Study Design and Sampling

**Figure 5:**
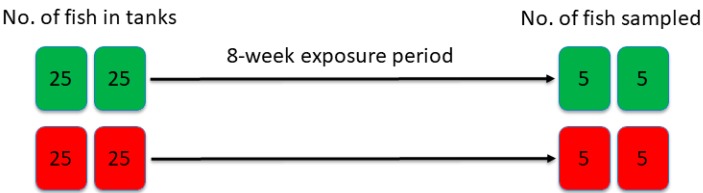
Design of the study on Atlantic salmon fed DON (5.5 mg/kg diet, red color) or no DON (controls, green color) for eight weeks. Five fish from each tank were sampled eight weeks after the start of the feeding trial.

This experiment was designed with two treatment groups (see Figure 5): a control group fed a standard pelleted feed (Spirit 3 mm, Skretting, Stavanger, Norway) with non-detectable levels of mycotoxins and a group offered the same feed coated with pure deoxynivalenol (DON) (Biopure standard DON; lot #06221Z, degree of purity 99.4%, Romer Labs, Tulln, Austria) to a level of 5.5 mg DON/kg feed. Each group comprised two tanks with 25 fish per tank. Five fish from each tank were sampled eight weeks after the start of the feeding trial, when mean weights for the control and DON fish were 123.2 g and 80.2 g, respectively. Thus, the weight gain of the DON group was only one third of the weight gain in the control group. The feed efficiency was approximately 0.8 and 1.3, respectively [11].

**Figure 6:**
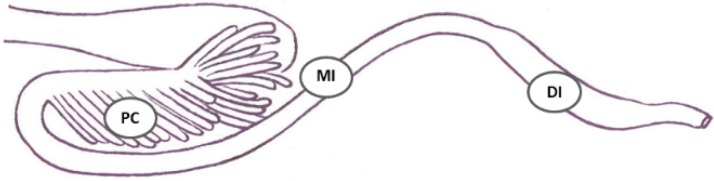
Sampling of intestinal tissues. Tissues from the pyloric ceca (PC), the mid-intestine (MI) and the distal intestine (DI) were collected on RNAlater and formalin. The drawing is a courtesy of Professor emeritus Inge Bjerkås, NMBU, Oslo, Norway.

Tissues from the pyloric ceca, the mid-intestine and distal intestine were collected on RNAlater and formalin (Figure 6). Tissues on RNAlater were stored cool for 24–48 h and then at −20 °C until further processing, while formalin-fixed tissues were routinely processed and embedded in paraffin after 24–48 h.

### 4.3. Gene Expression Analysis by Real-Time PCR

Nucleic acids from all intestinal tissues were extracted automatically on easyMag (bioMérieux, Marcy l’Etoile, France) using the generic protocol. The RNA concentration and purity were determined using a NanoDrop™ 2000 spectrophotometer (Thermo Scientific, Wilmington, DE, USA). Purity was assessed by determining the ratio of absorbance at 260 and 280 nm (A260/A280). All samples had a ratio between 1.80 and 2.08. The RNA was diluted to 50 ng/μL. The elimination of genomic DNA and the synthesis of cDNA from 500 ng RNA were performed with the QuantiTect Reverse Transcription Kit (QIAGEN, Hilden, Germany) according to the manufacturer’s recommendations using the included RT Primer Mix with an optimized blend of oligo-dT and random primers dissolved in water.

Real-time PCR was carried out using SsoAdvanced Universal SYBR Green Supermix (Bio-Rad, Hercules, CA, USA) with cDNA template corresponding to ~5 ng RNA in each reaction in a CFX384 Touch™ Real-Time PCR Detection System (Bio-Rad, Herkules, CA, USA) according to the producer’s instructions with 60 °C as annealing temperature and running 40 cycles.

The expression of markers for the following genes was analyzed by real-time PCR: claudin 25b, interleukin 1β (IL-1β), occludin, proliferating cell nuclear antigen (PCNA), suppressor of cytokine signaling 1 and 2 (SOCS1 and SOCS2), tricellulin, and finally elongation factor 1AB (EF1AB) as the reference gene [29] (see Table 2). All analyses were performed in triplicate, and a control lacking template for each master mix was always included in the experiments.

### 4.4. Histology and Immunohistochemistry

Sections from all intestinal tissues were cut at 3 μm and stained with hematoxylin and eosin (HE) or Alcian Blue (AB) and Periodic acid–Schiff (PAS) in combination, whereas proliferating cells were detected using a monoclonal mouse antibody against proliferating cell nuclear antigen (PCNA; M0879, Dako, Glostrup, Denmark) previously used in fish [33]. After deparaffinization, antigen retrieval, and inhibition as described elsewhere [34], the sections were incubated with horse serum diluted 1:100 in 5% bovine serum albumin (BSA) in a tris-buffer solution (TBS) for 20 min to prevent non-specific binding. The antibody against PCNA was diluted 1:5000 in 1% BSA/TBS before incubation for 60 min. The secondary antibody, horseradish peroxidase-labelled polymer conjugated to horse anti-mouse Ig, was diluted 1:100 in 1% BSA/TBS before incubation for 30 min, followed by incubation with Vectastain^®^ Elite ABC Reagent (PK-7100, Vector Laboratories, Burlingame, CA, USA) for 30 min and ImmPACT™ AEC (SK-4205, Vector Laboratories, Burlingame, CA, USA) for 5 min. The sections were thoroughly rinsed between each step except after the incubation with horse serum, counterstained with hematoxylin for 15 s and mounted with VectaMount™ AQ Aqueous Mounting Medium (H-5501, Vector Laboratories, Burlingame, CA, USA). Micrographs were captured with ACT-1 software (Nikon, Tokyo, Japan) using a digital camera D×1200 configured with a Leica DM4000 microscope.

### 4.5. Calculations and Statistical Analysis

Databases for the results from real-time PCR were established in Excel® for Windows, and statistical calculations and graphical presentation of gene expression were performed using Prism 7.0 software (GraphPad Software, La Jolla, CA, USA). Data were given as mean ± standard error of the mean (SEM) unless otherwise stated. The data for gene expression were analyzed for normality using the Shapiro–Wilk test. Data with normal distributions were further analyzed using the unpaired *t*-test, whereas non-normal data were analyzed using the Mann–Whitney U test. The significance level was set to 0.05.

## Figures and Tables

**Table 1:** Relative transcription levels normalized to reference gene EF1AB in the intestine of salmon fed DON (5.5 mg/kg feed) or no DON (controls) for eight weeks.

	Pyloric Ceca		Mid-Intestine		Distal Intestine	
	Control	DON	Control	DON	Control	DON
IL-1β	1 ± 0.27 ^a^	0.96 ± 0.40	1 ± 0.22 ^a^	0.94 ± 0.13	1 ± 0.21 ^a^	1.45 ± 0.25
SOCS1	**1 ± 0.23**	1.74 ± 0.27 *	1 ± 0.20 ^a^	0.99 ± 0.29	**1 ± 0.37 ^c^**	6.24 ± 2.17 *^,a^
SOCS2	1 ± 0.29	1.49 ± 0.28	1 ± 0.16 ^a^	2.26 ± 0.63	**1 ± 0.17**	7.19 ± 1.01 *
Claudin 25b	1 ± 0.14	1.25 ± 0.57	**1 ± 0.22 ^a^**	0.49 ± 0.09 *	**1 ± 0.20 ^a^**	0.27 ± 0.05 *^,a^
Occludin	**1 ± 0.10**	0.72 ± 0.32 *	**1 ± 0.10 ^a^**	0.60 ± 0.26 *^,b^	**1 ± 0.22**	0.50 ± 0.07 *
PCNA	**1 ± 0.24**	2.23 ± 0.31 *	**1 ± 0.16 ^a^**	2.98 ± 0.47 *	**1 ± 0.09**	1.70 ± 0.19 *
Tricellulin	1 ± 0.12	1.34 ± 0.54	**1 ± 0.17 ^a^**	0.32 ± 0.06 *	**1 ± 0.11**	0.49 ± 0.09 *

The data for the DON group are presented as mean ± standard error of the mean (SEM) relative to the mean ± SEM for the control group for the same intestinal segment; * Significant differences (unpaired *t*-test or Mann–Whitney U test, *p* < 0.05) between the experimental groups in the same intestinal segment. The values that differ significantly are highlighted in **bold** text. *n* = 10 for each group/gene/intestinal segment unless otherwise stated: ^a^: *n* = 9, ^b^: *n* = 8, and ^c^: *n* = 7.

**Table 2:** Primers for gene expression analyses.

Target	Gene Sequence 5→3′	Reference
Claudin 25b	F-CCTGTAAGAGGGGTCCATCAR-TGACACATGTTCTGCCCTGT	[27]
IL-1β	F-GCTGGAGAGTGCTGTGGAAGAR-TGCTTCCCTCCTGCTCGTAG	[30]
Occludin	F-GACAGTGAGTTCCCCACCATR-ATCTCTCCCTGCAGGTCCTT	[28]
PCNA	F-TGAGCTCGTCGGGTATCTCTR-GTCCTCATTCCCAGCACACT	[31]
SOCS1	F-TTCTTGATCCGGGATAGTCGR-TGTTTCCTGCACAGTTCCTG	[23]
SOCS2	F-CACTGCCAACGAAGCCAAAGAGATR-CAAACTGCTTCAGCTTGGGCTTGA	[23]
Tricellulin	F-GGATGCCATGATGGGTAAACR-AGGAAGGCTGGGTCACTCTT	[28]
EF1AB	F-TGCCCCTCCAGGATGTCTACR-CACGGCCCACAGGTACTG	[32]
